# ISPAD, bridging clinical expertise and AI for autoimmune-related pernicious anemia diagnosis

**DOI:** 10.3389/fimmu.2026.1700751

**Published:** 2026-04-28

**Authors:** Nora Boumela, Guillaume Cadiot, Farid Chaoui

**Affiliations:** 1 Department of Electronics, University of Batna 2, Batna, Algeria; 2 Departement of Electronics/LDCCP lab., Ecole Nationale Polytechnique, Algiers, Algeria; 3 Hepato-Gastroenterology and Digestive Oncology Department, Reims University Hospital Center, Reims, France; 4 Faculty of Medicine, University of Algiers, Algiers, Algeria

**Keywords:** AI-assisted diagnosis, B12 deficiency, fundic atrophy, OLGA classification, pernicious anemia

## Abstract

**Background:**

Pernicious anemia (PA) is a severe clinical consequence of autoimmune gastritis. It results from immune-mediated damage to gastric parietal cells in the oxyntic mucosa. This process leads to intrinsic factor deficiency and subsequent vitamin B_12_ malabsorption. This complex autoimmune response, combined with non-specific clinical manifestations, generates substantial diagnostic uncertainty. This uncertainty frequently results in misdiagnosis or delayed diagnosis and, consequently, multiple adverse outcomes, including irreversible neurological complications.

**Methods:**

To address the intrinsic diagnostic uncertainty of PA—arising from heterogeneous, graded, and often discordant clinical, histological, immunological, and biochemical information—we developed ISPAD (Intelligent System for Pernicious Anemia Diagnosis), an explainable AI–based probabilistic framework to integrate this information, producing a probability estimate of pernicious anemia that reflects clinical reasoning rather than rigid diagnostic thresholds.

**Results:**

ISPAD was examined using a series of published diagnostically challenging cases reflecting real-world complexity. These cases included antibody assay interference, hemolysis-masked macrocytosis, seronegative presentations, and cancer-associated atrophy. Across cases, the system generated a continuous and adaptable probabilistic assessment of pernicious anemia. This assessment relied on dynamic, context-dependent integration of available information and illustrated the potential for formalizing complex diagnostic reasoning.

**Conclusion:**

ISPAD illustrates how explainable artificial intelligence can formalize expert reasoning in autoimmune-related pernicious anemia. By integrating heterogeneous and often discordant information into a transparent probabilistic framework, this proof-of-concept approach provides a structured approach to diagnostic reasoning, particularly in complex or atypical situations.

## 1 Introduction

Pernicious anemia (PA) is a severe clinical consequence of autoimmune gastritis, a chronic immune mediated disorder affecting the oxyntic mucosa of the gastric fundus and corpus (1–3). In this condition, the immune system targets gastric parietal cells (4, 5), which are responsible for the secretion of hydrochloric acid and intrinsic factor (IF), a glycoprotein essential for vitamin B_12_ absorption (1, 5–9).

As the disease progresses, immune-mediated destruction of parietal cells leads to achlorhydria and IF deficiency. The absence of gastric acidity impairs iron absorption (10–14), whereas IF deficiency prevents adequate vitamin B12 uptake. This results in vitamin B12 deficiency, which underlies the megaloblastic anemia characteristic of pernicious anemia with potentially severe clinical consequences (15–21).

Despite advances in pathophysiological understanding, diagnosing pernicious anemia remains inherently challenging in clinical practice. This difficulty arises from multiple sources of uncertainty, including symptomatic complexity, non-specific clinical manifestations, and variable or discordant biomarkers, which often hinder differential diagnosis (22–26).

Although upper gastrointestinal endoscopy with systematic biopsy sampling remains the reference standard for confirming autoimmune gastritis in patients with suspected pernicious anemia, diagnostic interpretation relies on an appropriate clinical context and a sufficient pre-test probability. In routine clinical practice, early or atypical presentations (27–29), or inconclusive serological or biochemical data, may complicate the overall interpretation of histological findings, even when endoscopic evaluation is performed according to current recommendations (22, 30).

This diagnostic complexity, compounded by limited disease prevalence and reduced clinical awareness, frequently leads to misdiagnosis or delayed diagnosis (22, 30, 31), exposing patients to serious complications including neurological deficits and irreversible nerve damage (32–35). In addition, the underlying autoimmune gastritis is associated with an increased risk of gastric malignancy. These considerations underscore the importance of early and accurate diagnosis of this often misunderstood condition (31).

Artificial intelligence (AI) systems have rapidly emerged as promising tools in clinical research, with growing interest in their potential to assist clinicians in the analysis of complex clinical situations (36–44). Recent advances include the development of intelligent systems for anemia assessment (45), highlighting the relevance of computational approaches in hematological disorders.

Among these, AI models incorporating fuzzy logic principles are particularly well suited to diagnostic situations characterized by non-binary, graded, and partially discordant information (46–55). These models allow probabilistic handling of overlapping clinical and histological manifestations, graded interpretation of borderline biological biomarkers, and consideration of variability in immunological responses. These approaches provide a conceptual framework to formalize expert clinical reasoning under uncertainty, a challenge that is central to the diagnosis of autoimmune-related pernicious anemia.

Building on these principles, we developed ISPAD (Intelligent System for Pernicious Anemia Diagnosis), an explainable AI-based probabilistic framework designed to formalize structured diagnostic reasoning in autoimmune-related pernicious anemia.

ISPAD integrates histological (56–59), immunological, and biochemical information within a fuzzy logic framework to generate a probabilistic assessment of pernicious anemia. Although autoimmune gastritis is associated with an increased risk of gastric neuroendocrine tumors, these lesions represent late complications rather than primary diagnostic determinants of pernicious anemia; accordingly, ISPAD focuses on the diagnostic assessment of pernicious anemia rather than oncological risk stratification.

We applied ISPAD to published diagnostically challenging case reports reflecting real-world complexity. These cases included seronegative presentations, discordant diagnostic findings, borderline biochemical results, and conditions mimicking autoimmune-related pernicious anemia, including malignancy-associated gastric atrophy (60–65). This proof-of-concept analysis illustrates the system’s capacity to generate probabilistic estimates that are clinically coherent and internally consistent in ambiguous diagnostic scenarios. The objective of this work is not to propose a clinically validated tool but to formalize and make explicit the reasoning processes underlying complex diagnostic situations.

## 2 OLGA and pernicious anemia diagnosis: *key considerations*

Several classification systems are available for the assessment of chronic gastritis, including the Operative Link on Gastritis Assessment (OLGA) system (57), the Sydney system, and the Kimura–Takemoto classification (66). These frameworks differ in their criteria for evaluating the severity, topography, and histopathological features of gastric mucosal damage. In ISPAD, we adopted the OLGA staging system because its structured and reproducible approach allows a standardized assessment of both the severity and distribution of gastric atrophy (56–58), parameters that are central to the diagnostic reasoning in pernicious anemia. Importantly, OLGA is used without modification and strictly according to its original definition. By leveraging OLGA staging, ISPAD distinguishes between predominant antral atrophy, most commonly associated with Helicobacter pylori–related gastritis (67, 68), and corpus–fundus–predominant atrophy, which is strongly linked to autoimmune gastritis and the development of pernicious anemia (2, 5). This distinction provides a robust histopathological framework for subsequent probabilistic interpretation within the probabilistic framework.

### 2.1 Distinction between atrophy types and clinical interpretation

Fundic-predominant atrophy is strongly associated with autoimmune gastritis and represents the histopathological substrate most closely linked to pernicious anemia (5, 9). In contrast, antral atrophy is typically non-specific and most frequently observed in Helicobacter pylori–related gastritis (58, 68). The OLGA classification system provides a standardized framework for assessing the severity and topographic distribution of gastric atrophy.

In ISPAD, OLGA staging is conceptualized as a matrix in which each cell *T_ij_*corresponds to the intersection between antral atrophy (rows *i* = 0*,…*,3) and corpus/fundus atrophy (columns *j* = 0*,…*,3). This representation preserves the original OLGA framework and facilitates clinical interpretation of combined atrophy patterns. In consultation with gastroenterologists, cells characterized by isolated corpus–fundus atrophy (*T*_01_, *T*_02_, *T*_03_), highlighted in dark gray in **Table 1**, are considered highly compatible with autoimmune gastritis and pernicious anemia (5, 9). These patterns reflect the classical corpus-restricted atrophy that defines autoimmune-related pernicious anemia. Cells *T*_12_ and *T*_13_, highlighted in light gray, represent fundic-predominant atrophy with concomitant antral involvement. These configurations are less typical but clinically relevant, as autoimmune gastritis and pernicious anemia may coexist with antral atrophy related to H-pylori infection (58, 68) or other gastric conditions, particularly in advanced or long-standing disease. Accordingly, these patterns are not excluded but assigned a lower diagnostic weight within the probabilistic framework.

**Table 1:** OLGA staging table (56, 57) and its clinical interpretation for autoimmune-related pernicious anemia.

			Corpus/fundus atrophy		
		No (score 0)	Mild (score 1)	Moderate (score 2)	Severe (score 3)
Antrum atrophy	No (score 0)	STAGE 0	STAGE I	STAGE II	STAGE II
	Mild (score 1)	STAGE I	STAGE II	STAGE II	STAGE III
	Moderate (score 2)	STAGE II	STAGE II	STAGE III	STAGE IV
	Severe (score 3)	STAGE III	STAGE III	STAGE IV	STAGE IV

Cells shaded in gray highlight histopathological patterns suggestive of autoimmune-related pernicious anemia. Dark-gray cells indicate the most typical histopathological patterns of autoimmune related pernicious anemia, characterized by predominant corpus/fundus atrophy with sparing of the antral mucosa. Light-gray cells represent less typical but clinically plausible situations in which predominant corpus/fundus atrophy coexists with limited antral involvement. Other OLGA configurations are not excluded but are associated with lower probability estimates within the probabilistic framework.

Importantly, OLGA staging alone is insufficient for diagnosing pernicious anemia. In ISPAD, histological patterns are interpreted in conjunction with immunological parameters (autoantibodies) and biochemical markers (vitamin B12 and ferritin) to reflect real-world diagnostic reasoning and avoid rigid rule-based exclusion (20, 40).

Notably, the absence of a given OLGA configuration among the highlighted cells does not imply exclusion of pernicious anemia. Rather, these patterns reflect differing degrees of compatibility, and pernicious anemia may still occur in other OLGA stages, particularly in advanced or complex clinical contexts. Autoimmune gastritis may occur in a non-atrophic stage corresponding to OLGA 0 (*T*_00_), in which atrophy may not yet be detectable or may be histologically missed due to sampling limitations. While this markedly reduces the estimated probability of pernicious anemia, ISPAD does not categorically exclude its possibility and may generate intermediate probability estimates in rare but clinically plausible situations when immunological and biochemical features are strongly concordant. As with any probabilistic estimate, such intermediate values do not constitute diagnostic confirmation and must be interpreted in the overall clinical context. In OLGA 0 configurations, particular caution is therefore warranted. The *T*_11_ configuration corresponds to mild and symmetric antral and corpus atrophy, a pattern that lacks the oxyntic predominance characteristic of autoimmune gastritis. This profile is frequently observed in non–autoimmune chronic gastritis, particularly in Helicobacter pylori–associated gastritis, in which atrophy typically begins in the antrum and may progressively extend to the corpus, as well as in settings related to aging or environmental factors. Owing to this lack of topographic and immunological specificity, *T*_11_ does not have sufficient discriminative value to be considered a specific histopathological pattern for autoimmune gastritis or pernicious anemia and was therefore assigned a lower, non-zero diagnostic weight within the probabilistic framework of ISPAD, rather than being excluded.

Accordingly, in this framework, antral atrophy is not interpreted as a diagnostic criterion for autoimmune gastritis, but as a subordinated discriminative parameter, used to modulate the estimated probability of autoimmune-related pernicious anemia. The next section introduces the AI-based inference framework underlying ISPAD and explains how it formalizes clinical reasoning under diagnostic uncertainty.

## 3 Intelligent system for pernicious anemia diagnosis

ISPAD was designed as an explainable probabilistic framework to estimate the probability of autoimmune related pernicious anemia by integrating heterogeneous diagnostic information. In real-world clinical practice, diagnostic evidence is often graded, incomplete, or partially discordant. To accommodate this complexity while preserving interpretability, ISPAD employs fuzzy logic concept—a framework that models uncertainty through expert-defined rules rather than opaque and black-box machine learning models that sacrifice interpretability.

### 3.1 System architecture and inference mechanism

The architecture of ISPAD integrates histological, immunological, and biochemical parameters within a unified fuzzy inference framework to generate transparent probabilistic estimates. Rather than relying on isolated criteria, this multimodal approach allows for diagnostic compensation when individual markers are absent or discordant, effectively mirroring real-world clinical reasoning.

Clinically relevant parameters are first translated into interpretable linguistic categories (e.g., Low, Normal, and High) using predefined Membership Functions (MFs). These linguistic inputs are then combined through expert-derived IF–THEN rules. The fuzzy inference mechanism applies this rule base to produce an overall assessment, which is subsequently defuzzified into a final probability estimate (0%–100%) of autoimmune related PA. This architecture enables ISPAD to account for borderline values, partial concordance, and conflicting diagnostic signals while preserving full traceability of the reasoning process (69). Detailed technical specifications are provided in (**Supplementary Tables S1**, **S2**; **Supplementary Text S1**).

#### 3.1.1 ISPAD parameters

The input parameters of ISPAD were selected based on their clinical relevance, availability in routine practice, and direct involvement in the pathophysiology of autoimmune-related pernicious anemia. Parameter selection and their relative influence within the fuzzy inference process were established in close collaboration with gastroenterology specialists from a tertiary referral center (CHU of Reims) to reflect real-world diagnostic reasoning.

ISPAD integrates six diagnostic parameters grouped into three complementary domains:

##### 3.1.1.1 Histological parameters (atrophic gastritis)

- OLGA stage (0–IV). OLGA staging provides a standardized assessment of the severity and topographic distribution of gastric atrophy. Fundic-predominant atrophy constitutes the histopathological substrate most strongly associated with autoimmune gastritis and pernicious anemia.
- Antral atrophy score (0-3). Antral atrophy is not a diagnostic criterion for autoimmune gastritis but serves as a discriminative parameter. In ISPAD, it modulates diagnostic probability by helping distinguish autoimmune-related fundic atrophy from other etiologies, particularly Helicobacter pylori–associated gastritis. Concomitant antral involvement does not exclude pernicious anemia, especially in advanced or long-standing disease (70).

##### 3.1.1.2 Immunological parameters (autoimmune biomarkers of atrophic gastritis)

- Anti-parietal cell antibodies (APCA). APCA are sensitive but poorly specific markers of autoimmune gastritis. Their presence supports an autoimmune process but cannot independently confirm pernicious anemia (9, 71, 72).
- Anti-intrinsic factor antibodies (AIFA). AIFA exhibit high specificity for pernicious anemia and play a dominant diagnostic role when present (4, 9, 71). However, limited sensitivity requires interpretation in conjunction with histological and biochemical data.

##### 3.1.1.3 Biochemical parameters (factors involved in red blood)

- Serum vitamin B12. Low vitamin B12 levels reflect impaired absorption due to intrinsic factor deficiency but are not pathognomonic for pernicious anemia. Borderline or normal values may occur due to supplementation, assay interference, or early disease.
- Serum ferritin. Ferritin reflects iron stores and may reveal iron deficiency secondary to achlorhydria, as gastric atrophy impairs dietary iron conversion (*Fe*^3+^ → *Fe*^2+^) (11–14). Its interpretation requires clinical context, as inflammatory states may elevate ferritin independently of iron status.

#### 3.1.2 Formal definition of inputs and output

For clarity, the formal definition of ISPAD inputs and output is provided below before detailing the multimodal integration and inference mechanisms.

As we have seen above, to address the diagnostic complexity of PA, the ISPAD framework integrates six clinically relevant parameters forming the system input vector, as defined in Equation 1:

(1) $$x=[x_{1},x_{2},\ldots ,x_{6}]\in {\mathbb{R}}^{6}$$

where *x_i_*corresponds to the specific biomarkers *x_i_*=OLGA,Antrum, APCA,AIFA, B12, Ferritin.

The system output is a continuous pernicious anemia probability (PAP), expressed as a percentage (0%–100%), representing the estimated likelihood of autoimmune-related pernicious anemia based on integrated diagnostic evidence. The PAP is obtained through a non-linear mapping, as described in Equation 2:

(2) PAP=f(x)∈[0,100]

**Figure 1:**
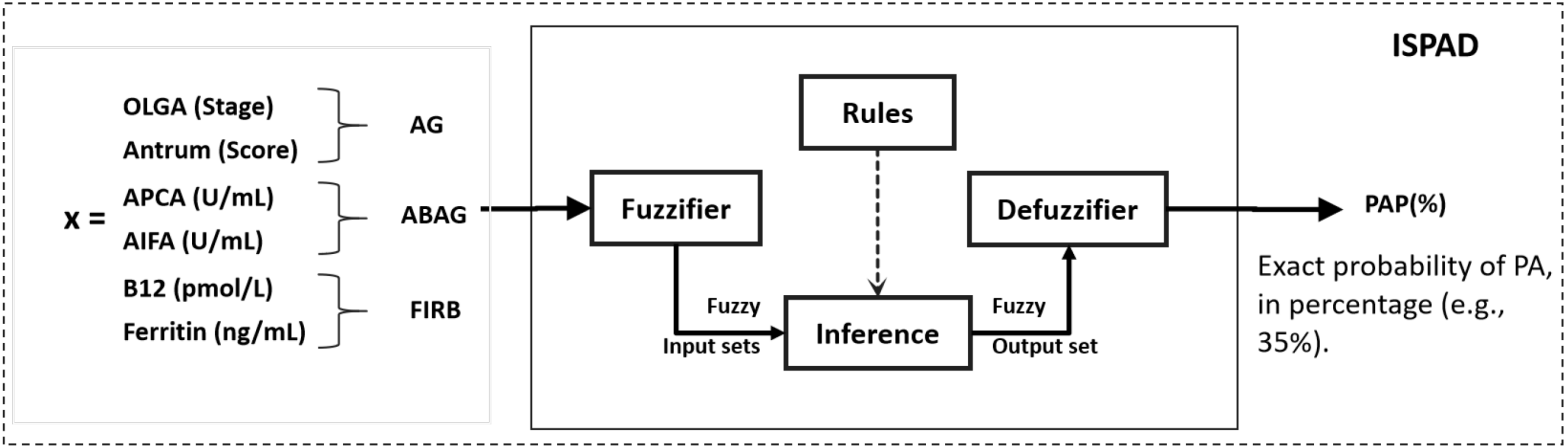
Architecture of the intelligent system for pernicious anemia diagnosis (ISPAD). ISPAD integrates histological (OLGA staging, antral atrophy), immunological (APCA, AIFA), and biochemical parameters (vitamin B_12_, ferritin) within a fuzzy inference framework. Input variables are fuzzified, combined using expert-defined rules, and defuzzified to generate a continuous Pernicious Anemia Probability assessment (PAP, %).

Here, $f:{\mathbb{R}}^{p=6}\to [0,100]$ represents the diagnostic reasoning framework that integrates histological, immunological, and biochemical information through clinical rule-based inference and uncertainty modeling (**Figure 1**).

Accordingly, ISPAD is not intended to redefine or replace established diagnostic criteria for pernicious anemia but to formalize expert reasoning under uncertainty in complex or atypical clinical contexts.

A schematic overview of the ISPAD architecture and inference process is shown in **Figure 1**. Technical definitions of membership functions, inference rules, and mathematical details are provided in **Supplementary Tables S1**, **S2**, **Supplementary Text S1**.

#### 3.1.3 Membership functions

Within the ISPAD framework, each input parameter is interpreted in a graded manner through predefined fuzzy membership functions, allowing continuous clinical and biological values to be mapped to meaningful linguistic categories (e.g., low, borderline, and high). This approach reflects the inherent uncertainty and variability of diagnostic markers in routine practice and avoids rigid threshold-based interpretation.

The definition and calibration of membership functions were established in collaboration with clinical experts. Detailed technical specifications are provided in **Supplementary Tables S1**, **S2**.

#### 3.1.4 Rule basis

The core of the ISPAD inference process relies on an expert-defined rule base composed of IF–THEN rules that formalize clinical diagnostic reasoning. Each rule combines histological, immunological, and biochemical information to estimate the likelihood of autoimmune-related PA.

For example, a typical rule may reflect the following reasoning:

- IF OLGA staging is Mild, the Antral mucosa is *Spared*, Intrinsic Factors antibodies are Present, and Vitamin B12 levels are Low, THEN the PAP is Very High.
- Conversely, isolated antibody positivity or biochemical abnormalities without compatible histology are assigned lower probability levels.

An illustrative example of such a rule is provided here for clarity, whereas the mathematical inference framework is detailed in **Supplementary Text S1**. The complete executable rule base is provided in the native.fis file included in the **Supplementary Material**.

This rule-based structure allows ISPAD to encode clinically meaningful interactions between parameters, including compensatory and synergistic effects, while preserving transparency and interpretability.

### 3.2 Medical expert-driven rule development

The ISPAD rule base was co-developed in close collaboration with gastroenterology and digestive oncology experts, led by Prof. Guillaume Cadiot, and is based on a set of expert-defined IF–THEN rules integrating histological, immunological, and biochemical information.

#### 3.2.1 Clinical thresholds

- APCA positivity (≥ 40 U/mL), AIFA positivity (≥ 10 U/mL), and OLGA staging (Stg0–StgIV) aligned with international guidelines.
- Functional markers such as vitamin B12 (*<* 200 pg/mL=“Low”) and ferritin (*<* 30 µg/L=“Low”) were interpreted using clinically validated thresholds.

#### 3.2.2 Pathophysiological synergies

Corpus–fundus–predominant atrophy patterns (*e.g.*, T01–T03 in **Table 1**) are prioritized, as they are classically associated with autoimmune gastritis and help distinguish it from H. pylori–associated atrophy.

- Antibody interactions are modeled to reflect the higher specificity of AIFA and the complementary role of APCA, particularly in seronegative or serologically discordant contexts.
- Metabolic dependencies are incorporated to account for borderline vitamin B12 levels and iron deficiency dynamics.

#### 3.2.3 Handling diagnostic ambiguities

Diagnostic uncertainty is intrinsic to pernicious anemia, as histological, immunological, and biochemical findings may be absent, borderline, or discordant across different stages of the disease. In routine clinical practice, this uncertainty commonly arises from assay interference, incomplete or non-representative histological sampling, and the non-binary behavior of biomarkers near clinical decision thresholds.

Within ISPAD, such ambiguity is not resolved through rigid inclusion or exclusion rules but is instead propagated through the inference process in a graded manner. By translating each input parameter into degrees of membership and combining them across multiple rules, the system allows partial or conflicting evidence to modulate—rather than determine—the final probability estimate. This design ensures that diagnostically plausible situations are not artificially excluded when individual markers are inconclusive.

Accordingly, ISPAD explicitly accounts for recurrent and clinically relevant sources of uncertainty that directly emerge from the modeled inputs, while remaining grounded in expert-defined diagnostic reasoning.

- Diagnostic ambiguity related to serology is addressed through graded handling of isolated, discordant, or negative antibody findings (including seronegative presentations).
- Analytical and biological uncertainty, such as assay interference (e.g., AIFA^+^ despite normal vitamin B_12_ levels) or borderline biomarker values, is modeled using graded transitions rather than fixed thresholds.
- Uncertainty at classification boundaries is further captured through non-linear membership functions (e.g., trapezoidal, triangular, and Gaussian), allowing smooth transitions between diagnostic categories.

This clinically grounded framework ensures that ISPAD captures the complex interplay between anatomical (AG), immunological (ABAG), and metabolic (FIRB) parameters. By embedding clinical expertise within a fuzzy logic-based framework, the rule base overcomes key limitations of conventional diagnostic criteria—such as rigid biochemical thresholds and single-marker reliance—while preserving full adaptability to atypical, borderline, and complex diagnostic scenarios, as demonstrated in **Table 2**.

**Table 2:** Partial rule table used by ISPAD for pernicious anemia probability (PAP), showing probability estimation from extremely low (EL) to extremely high (EH).

OLGA	Antrum	APCA	AIFA	B12↑ Fer.↑	B12↑ fer.↓	B12↓ fer.↑	B12↓ fer.↓	Clinical rationale
0	0	N	N	EL	EL	EL	EL	No atrophy
	0	N	P	VL2	L1	MH	MH	Isolated AIFA+
	0	P	N	EL	EL	EL	EL	Isolated APCA+
	0	P	P	VL2	L2	MH	MH	Double antibodies
	All other antrum values (1-3) are non-existent for OLGA 0							
1	0	N	N	M	MH	H1	H2	Fundic-predominant
	0	N	P	VH1	VH2	VH2	VVH	Fundic-predominant with AIFA+
	0	P	N	H1	H2	VH1	VH2	Fundic-predominant with APCA+
	0	P	P	VH2	VVH	VVH	EH	With autoimmunity
	1	N	N	EL	EL	VVL	VL1	Mixed pattern
	1	N	P	L1	L2	L2	M	Mixed pattern with AIFA+
	1	P	N	VL1	VL2	L1	L2	Mixed pattern with APCA+
	1	P	P	L2	M	M	MH	Mixed pattern with autoimmunity
	Antrum ≥ 2 is non-existent for OLGA 1							
2	0	N	P	VH2	VVH	VVH	EH	Moderate fundic atrophy
	1	P	N	M	H1	H2	H2	With antral involvement
	2	N	N	EL	VL1	VL2	L1	Balanced atrophy
	Antrum=3 is non-existent for OLGA 2							
3	1	N	N	M	MH	H1	H2	Fundic-dominant
	2	P	P	L2	M	M	H1	With antral atrophy
	3	N	N	EL	VVL	VL1	VL2	Antrum-predominant
	Antrum=0 is non-existent for OLGA 3							
4	2	P	P	L2	M	M	VH1	Severe atrophy
	3	N	N	EL	VVL	VL1	VL2	End-stage
				*Antrum ≤ 1 is non-existent for OLGA 4*				

Clinically impossible OLGA/Antrum combinations (0,1–3; 1,2–3; 2,3; 3,0; 4,0–1) are excluded based on histopathological incompatibility according to the OLGA staging system (56). For reasons of space, only selected combinations are shown; the complete executable rule base is provided in the native.fis file included in the **Supplementary Material**. Risk categories reflect probabilistic estimates rather than categorical diagnoses and are intended to support, not replace, expert clinical judgment.

## 4 ISPAD evaluation

To better understand the system’s behavior and ensure its clinical coherence, we employed surface plots to visualize the relationships between key input parameters and the model output. These graphical analyses illustrate how graded changes in histological, immunological, and biochemical variables influence the estimated probability of pernicious anemia. Rather than serving as a technical performance assessment, these visualizations provide an interpretable representation of the system’s internal reasoning, highlighting clinically plausible trends and decision pathways across different diagnostic configurations.

### 4.1 Analysis of PAP across ISPAD inputs

**Figure 2:**
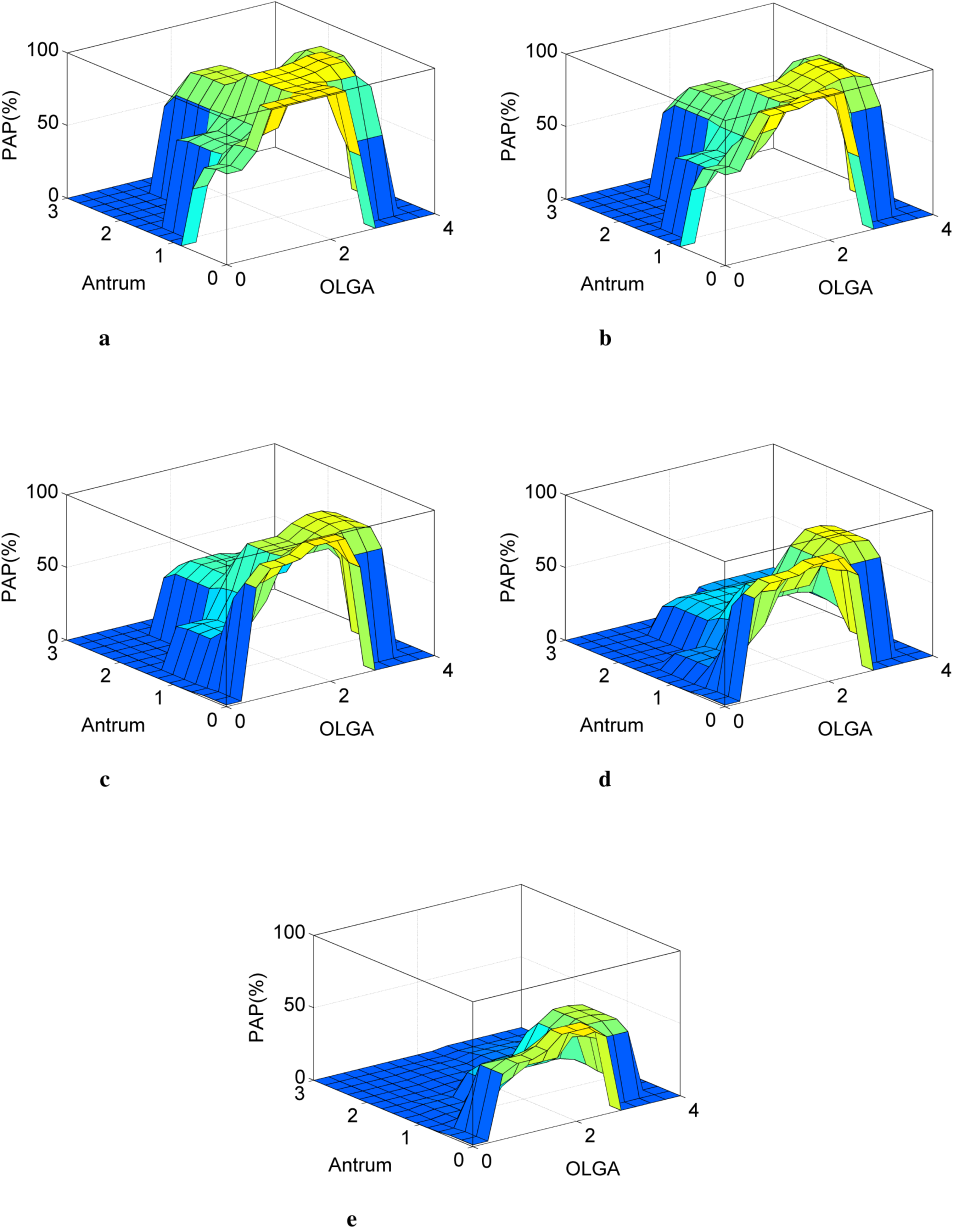
Impact of AG parameters on the system output: different configurations. **(a)** x=[OLGA, Antrum, APCA^++^, AIFA^++^, B12↓, Ferritin↓], **(b)** x=[OLGA, Antrum, APCA^−−^, AIFA^++^, B12↓, Ferritin↓], **(c)** x=[OLGA, Antrum, APCA^++^, AIFA^−−^, B12↓, Ferritin↓], **(d)** x=[OLGA, Antrum, APCA^−−^, AIFA^−−^, B12↓, Ferritin↓], **(e)** x = [OLGA, Antrum, APCA^−−^, AIFA^−−^, B12↑, Ferritin↑].

**Figures 2a–d** illustrate the ISPAD system’s output as a function of AG category parameters, with variations in other inputs to evaluate their influence on the estimated probability of pernicious anemia.

Remark: For clinically implausible parameter combinations—specifically OLGA stage IV with absent or minimal antral atrophy (Antrum = 0 or 1), or OLGA stage 0 with moderate to severe antral atrophy (Antrum = 1–3), corresponding to incompatible configurations in the OLGA matrix—the system constrains the estimated PAP to zero (visible in the bottom corners of **Figure 2a**). This behavior reflects strict adherence to histopathological compatibility as defined by the OLGA staging framework, rather than an assumption of negligible disease probability, and ensures internal consistency between the OLGA classification matrix and the model’s probabilistic outputs. (**Figure 2a**).

#### 4.1.1 Scenario 1: positive ABAG and low FIRB parameters

When autoimmune biomarkers are positive (APCA^+^, AIFA^+^) and functional deficiency markers are present (B12 ↓, Ferritin ↓), the ISPAD output surfaces display a marked probability peak over OLGA/Antrum configurations corresponding to isolated corpus–fundus atrophy with spared antrum. These maximal probability regions directly map to cells T_01_, T_02_, and T_03_ of the OLGA-based matrix (**Table 1**), which are highlighted in dark gray and represent the most typical histopathological pattern of autoimmune related pernicious anemia (PAP=≈ 97%). As antral involvement increases while corpus–fundus atrophy remains predominant, the probability peak progressively decreases. This attenuation is observed over configurations corresponding to cells T12 and T13, highlighted in light gray in **Table 1**. These cells represent fundic-predominant atrophy with limited antral involvement—patterns that remain clinically plausible for pernicious anemia but are less specific than isolated fundic atrophy. Importantly, the continuous decline of the probability peak across the surface plots mirrors the transition from dark gray to light gray cells in the OLGA matrix. This correspondence demonstrates that the fuzzy inference system does not impose arbitrary probability gradients but rather formalizes the graded histopathological interpretation embedded in **Table 1**. In other words, the surface representations constitute a continuous projection of the discrete OLGA-based classification matrix into a probabilistic space, preserving both the hierarchy and the clinical meaning of the underlying histopathological patterns. The highest probability plateaus correspond to the most specific OLGA configurations for autoimmune-related pernicious anemia, while progressively lower plateaus reflect decreasing diagnostic specificity as antral involvement increases.

Clinical insights derived from surface analysis.

- OLGA Stage 4 with Antrum Score 2 (T32): The estimated probability decreases, reflecting that severe corpus–fundus atrophy combined with moderate antral involvement complicates diagnostic specificity. Nevertheless, the probability remains high, consistent with advanced autoimmune-related disease.
- OLGA Stage 4 with Antrum Score 3 (T33): The probability further declines, indicating that equal or antrum-predominant atrophy substantially reduces the likelihood of pernicious anemia, although it does not fully exclude it.
- OLGA Stage 0 with Antrum Score 0 (T00): Although the absence of histological atrophy generally lowers the estimated probability of pernicious anemia, ISPAD does not categorically exclude the diagnosis in this configuration. In **Figure 2**, T00 may still be associated with non-negligible probability values when strong concordant immunological and biochemical signals are present. In particular, isolated intrinsic factor antibody (AIFA) positivity combined with marked vitamin B12 deficiency (e.g., case 3) raises the estimated probability to approximately 60%, despite preserved gastric histology. This behavior reflects clinically plausible scenarios of early autoimmune gastritis, focal oxyntic involvement, or sampling-related histological underestimation, in which pernicious anemia may precede detectable atrophy. This observation aligns with current pathological understanding and underscores ISPAD’s design philosophy: to estimate the probability of clinically relevant pernicious anemia rather than to exclude it based on a single histological criterion.

Importantly, this probabilistic behavior is consistent with the OLGA-based matrix (**Table 1**), where OLGA stage 0 is not treated as an exclusion criterion but as a configuration with low baseline probability that may be modulated upward by non-histological evidence.

#### 4.1.2 Scenario 2: APCA^−^/AIFA^+^ and low FIRB parameters

**Figure 2b** illustrates a clinically relevant scenario in which AIFA positivity (≥ 10 U/mL) and functional deficiency markers (B12↓, Ferritin↓) dominate the diagnostic assessment despite negative APCA (*<* 40 U/mL). In fundic-predominant configurations (OLGA 1–2, antrum 0–1; cells T10–T13), ≈95%, reflecting the high specificity (98.6%) of AIFA for pernicious anemia. Across the surface, PAP remains consistently higher in AIFA^+^ scenarios than in APCA^+^ scenarios, particularly as antral involvement increases. This behavior highlights the system’s prioritization of AIFA over APCA (near-pathognomonic profile), consistent with established diagnostic hierarchies. Clinically, this confirms that:

- AIFA testing should be prioritized when pernicious anemia is suspected;
- Negative APCA does not exclude PA when AIFA, OLGA patterns, and biochemical deficiency are concordant.

This case exemplifies how ISPAD preserves expert diagnostic reasoning by weighting antibody specificity and histological context rather than relying on isolated markers.

#### 4.1.3 Scenario 3: APCA^+^/AIFA*^−^*and low FIRB parameters

**Figure 2c** illustrates diagnostically relevant differences between isolated APCA positivity (APCA^+^/AIFA^−^) and the AIFA-positive configuration examined in case 2 (APCA^−^/AIFA^+^), under comparable biochemical conditions (low B12/ferritin). Key comparison between two fundus-predominant configurations highlights this effect:

- (OLGA 1, Antrum 0)
- APCA^+^/AIFA^−^: PAP ≈87%
- APCA^−^/AIFA^+^: PAP ≈95%
- Difference: 8% probability gap favoring AIFA positivity

Pattern analysis shows that, although both configurations initially yield high probabilities in fundus predominant atrophy:

- AIFA^+^ profiles maintain PAP values *>* 90% across OLGA stages 1–3 when the antrum is spared (Antrum 0–1).
- APCA^+^ profiles exhibit:
- a steeper probability decline with increasing antral involvement (≈−25%per antral stage vs. ≈−12% for AIFA^+^),
- 15%–20% lower PAP values in advanced stages (OLGA 3–4, Antrum ≥2).

##### 4.1.3.1 Clinical significance

1.Even under optimal histological conditions (OLGA 1, Antrum 0), isolated APCA positivity yields high but consistently lower PAP values compared with AIFA positivity, reflecting the higher specificity of AIFA (≈98.6% vs. ≈90%).

2.The divergence between APCA and AIFA profiles increases with:

- progressive antral involvement (ΔPAP ≈ +20% in favor of AIFA^+^ at Antrum 1),
- advanced OLGA stages (Δ PAP ≈ +25% at OLGA 3).

Overall, this case demonstrates ISPAD’s ability to model nuanced antibody–histology interactions and to reproduce the known diagnostic hierarchy between AIFA and APCA within a probabilistic framework, even when serological markers are discordant.

#### 4.1.4 Scenario 4: ABAG*^−^* and FIRB*↓* parameters

**Figure 2d** examines a clinically relevant scenario in which autoimmune biomarkers are negative (APCA^−^/AIFA^−^), whereas functional deficiency markers are altered (low vitamin B12 and ferritin). Compared with antibody-positive profiles, PAP values decrease overall, but remain substantially elevated.

(∼80%) in specific histopathological configurations. High PAP values are observed in regions characterized by fundus-predominant atrophy with spared or minimally involved antrum:

- OLGA Stage 2 with spared antrum (Antrum 0): Cells T02/T03 ((OLGA, Antrum) = (2,0)) show persistently high PAP, consistent with the classical histopathological pattern of autoimmune gastritis.
- OLGA Stage 3 with mild antral involvement (Antrum 1): Cell T13 shows a moderate probability decrease (∼76%), reflecting reduced diagnostic specificity as atrophy extends beyond the fundus.
- Advanced stages (OLGA ≥ 4, Antrum ≥ 2): PAP drops sharply (∼15%; T22/T33), as pan-gastric atrophy becomes atypical for pernicious anemia.

##### 4.1.4.1 Clinical implications

1.Seronegative pernicious anemia: Fundus-predominant atrophy combined with low FIRB parameters may indicate:

- early-stage disease (pre–antibody seroconversion),
- antibody levels below detection thresholds,
- false-negative serology due to assay limitations or intermittent antibody secretion.

2.Diagnostic nuance: The progressive involvement of the antrum is quantitatively associated with decreasing PAP, aiding differentiation between autoimmune-related pernicious anemia and other gastropathies.

This case highlights ISPAD’s ability to integrate histological and metabolic evidence to generate clinically plausible probability estimates, even in the absence of autoimmune serological markers.

#### 4.1.5 Scenario 5: ABAG*^−^*and normal FIRB parameters

In **Figure 2e**, we examine a scenario combining negative ABAG parameters (APCA^−^/AIFA^−^) with normal FIRB values (normal vitamin B12 and ferritin). While this configuration typically yields low probabilities of pernicious anemia (PA), two clinically relevant exceptions emerge.

- Isolated fundic atrophy ((*OLGA*, Antrum) = {(1,0),(2,0)}):
- Cells T01–T02 maintain moderate PAP values (50%–60%) despite negative serology.
- This configuration requires both complete antral sparing (Antrum = 0) and mild-to-moderate fundic atrophy (OLGA 1–2).
- Mild antral involvement ((3,1), T13):
- PAP declines to approximately 40%, compared with 50%–60% in isolated fundic atrophy.
- This progressive decrease suggests either:
- *H. pylori*–associated atrophy with focal fundic involvement, or
- alternative variants of autoimmune gastritis.

Interpretation: These residual probability estimates may reflect:

- Technical factors:
- false-negative antibody testing (anti-intrinsic factor assay sensitivity: 40%–60%),
- subthreshold antibody levels (pre-seroconversion phase).
- Biological factors:
- early-stage pernicious anemia preceding overt metabolic disturbances,
- non–PA etiologies mimicking autoimmune gastric patterns.

Overall, these case-based evaluations illustrate how ISPAD integrates histological patterns, immunological markers, and biochemical indicators to generate clinically coherent probability estimates across a wide range of real-world diagnostic scenarios. Rather than relying on abstract parameter interactions, the system’s behavior is best understood through its alignment with established histopathological patterns and clinically interpretable configurations, as formalized in the OLGA-based matrix and exemplified by the representative cases presented above.

### 4.2 Concluding remarks

Together, the case-based evaluations demonstrate how ISPAD integrates heterogeneous diagnostic information and remains sensitive to clinically meaningful interactions between histology, immunological markers, and functional deficiency parameters. Rather than relying on isolated criteria, the system resolves diagnostic ambiguities by reproducing expert reasoning across a spectrum of realistic and challenging clinical configurations. These findings support the robustness of ISPAD when applied to real-world diagnostic contexts, where information is often incomplete, graded, or partially discordant.

## 5 Case presentations

To evaluate ISPAD in real-world settings, we analyzed seven diagnostically challenging cases with confirmed pernicious anemia, selected to cover recurrent sources of diagnostic complexity: assay interference with apparently normal vitamin B_12_, dual-antibody positivity with hemolysis, histology-negative early-stage presentations, seronegative profiles, competing nutritional deficiencies, autoimmune comorbidity, and malignancy-associated gastric pathology (60–65).

For each case, we extracted ISPAD input parameters (OLGA stage, antral atrophy, APCA, AIFA, vitamin B_12_, and ferritin) from the source reports, and summarized the system output as the Pernicious Anemia Probability (PAP), as presented in **Table 3**. Detailed clinical narratives and complete diagnostic workups are provided in **Supplementary Text S2**.

Importantly, all diagnoses had been established prior to ISPAD analysis using standard clinical, biological, endoscopic, and histopathological criteria. ISPAD was therefore not used as a diagnostic tool, but as an evaluative framework to assess its ability to reproduce expert clinical reasoning and to generate coherent probabilistic estimates in complex or atypical presentations.

### 5.1 Case 1: AIFA^+^ with normal vitamin B_12_

#### 5.1.1 ISPAD analysis

Despite normal serum vitamin B_12_ and ferritin concentrations, ISPAD generated a very high Pernicious Anemia Probability (PAP = 87.6%). This estimate was primarily driven by strong anti-intrinsic factor antibody positivity and the presence of isolated corpus–fundus–predominant atrophy with antral sparing (OLGA stage II, antrum score 0), a histopathological configuration highly compatible with autoimmune related pernicious anemia. Normal vitamin B_12_ and ferritin values exerted only minimal down-weighting, consistent with known assay interference in intrinsic factor antibody–positive patients and the absence of iron deficiency at presentation.

#### 5.1.2 Clinical correlation

Following intramuscular cyanocobalamin therapy, the patient experienced rapid hematological recovery and marked improvement in neuropsychiatric symptoms. Lower-limb paresthesias resolved more gradually but ultimately subsided. This case illustrates ISPAD’s capacity to prioritize highly specific immunological and compatible histological features over potentially misleading biochemical parameters, reproducing expert clinical reasoning in an atypical diagnostic context.

### 5.2 Case 2: AIFA^+^ and APCA^+^ with autoimmune hemolytic anemia

#### 5.2.1 ISPAD analysis

ISPAD generated PAP of 99.1%, corresponding to the *Extremely High* output category. The estimate was primarily driven by the strong concordance of dual autoimmune serology (AIFA^+^ and APCA^+^) and advanced corpus-predominant atrophy (OLGA stage II). Severe vitamin B_12_ deficiency further reinforced the probability estimate, whereas elevated ferritin levels related to inflammation and recent transfusion history were appropriately interpreted within their clinical context.

Importantly, the presence of autoimmune hemolytic anemia and transfusion-related hematological disturbances did not reduce the PAP. ISPAD prioritized histological (OLGA stage and absence of antral atrophy) and immunological (AIFA/APCA) inputs, while interpreting biochemical markers within their clinical context. As a result, concomitant hematological processes did not bias the probabilistic assessment of pernicious anemia.

#### 5.2.2 Clinical correlation

The patient was treated with high-dose corticosteroids for autoimmune hemolytic anemia and parenteral vitamin B_12_ supplementation. This combined therapeutic approach resulted in rapid hematological recovery and progressive normalization of hemolysis markers. Vitamin B_12_ replacement was continued long term, with stabilization of hemoglobin levels and subsequent tapering of corticosteroid therapy.

This case illustrates ISPAD’s ability to preserve diagnostic specificity for pernicious anemia in the presence of overlapping autoimmune hematological disorders. By prioritizing autoimmune serology and compatible histopathological patterns over potentially confounding hematological phenomena, ISPAD reproduces expert clinical reasoning in complex autoimmune presentations.

### 5.3 Case 3: AIFA^+^ with pseudothrombotic microangiopathy

#### 5.3.1 ISPAD analysis

In this case, ISPAD generated an intermediate-to-high Pernicious Anemia Probability (PAP = 62.5%). The estimated probability was primarily driven by strong anti-intrinsic factor antibody (AIFA) positivity and severe vitamin B_12_ deficiency, both of which are highly specific markers of pernicious anemia.

In contrast, the absence of histological atrophy (OLGA stage 0 with preserved antral mucosa) appropriately moderated the final probability estimate, preventing overclassification. This behavior reflects ISPAD’s capacity to model early or non-atrophic stages of autoimmune gastritis, in which immunological and functional abnormalities may precede overt structural gastric changes.

It is important to note that autoimmune gastritis may occur in a non-atrophic stage corresponding to OLGA stage 0, in which oxyntic atrophy may be absent, focal, or histologically undetected due to sampling limitations. In such early or non-atrophic forms, the development of overt pernicious anemia is uncommon but remains clinically plausible in the presence of highly specific immunological markers and functional vitamin B_12_ deficiency.

Accordingly, ISPAD does not exclude the diagnosis in OLGA stage 0 but assigns an intermediate probability estimate when immunological and biochemical evidence is strongly concordant. This probabilistic behavior reflects both the rarity of pernicious anemia in non-atrophic autoimmune gastritis and the need to account for early or evolving disease stages without overclassification.

Importantly, confounding hematological manifestations related to pseudothrombotic microangiopathy did not influence the probabilistic output, as ISPAD relies exclusively on histological, immunological, and biochemical input parameters rather than secondary hematological consequences of vitamin B_12_ deficiency.

#### 5.3.2 Clinical correlation

The patient was treated with parenteral cyanocobalamin, resulting in complete hematological recovery, resolution of schistocytosis, and normalization of platelet counts within six weeks. This rapid and sustained response confirmed the diagnosis of AIFA-positive pernicious anemia despite the absence of gastric atrophy on histology.

This case illustrates ISPAD’s ability to identify pernicious anemia in early or histology-negative stages by appropriately prioritizing highly specific immunological markers and functional vitamin B_12_ deficiency, while avoiding misclassification driven by atypical hematological presentations such as pseudothrombotic microangiopathy.

### 5.4 Case 4: seronegative (AIFA*^−^* APCA*^−^*) with iron overload

#### 5.4.1 ISPAD analysis

In this seronegative presentation (AIFA^−^/APCA^−^), ISPAD generated a high Pernicious Anemia Probability (PAP = 77.5%). The estimated probability was primarily driven by the presence of corpus–fundus–predominant gastric atrophy (OLGA stage II, antrum score 0) combined with severe vitamin B_12_ deficiency.

Despite the absence of autoimmune antibodies, the histological pattern was highly compatible with autoimmune gastritis and therefore played a dominant role in the probability estimate. Marked hyperferritinemia did not reduce the PAP, as ISPAD interprets ferritin levels in a pathophysiological context and does not consider isolated iron overload as an exclusion criterion for pernicious anemia.

This behavior reflects ISPAD’s ability to identify seronegative forms of pernicious anemia by prioritizing compatible histological and metabolic patterns over immunological negativity.

#### 5.4.2 Clinical correlation

Parenteral vitamin B_12_ supplementation resulted in rapid hematological recovery, with normalization of hemoglobin and platelet counts. Ferritin levels progressively declined over follow-up, confirming that iron overload was secondary to ineffective erythropoiesis rather than a primary iron metabolism disorder.

This case illustrates ISPAD’s capacity to support the diagnosis of pernicious anemia in seronegative patients with misleading biochemical profiles, by reproducing expert clinical reasoning based on integrated histological and metabolic evidence.

### 5.5 Case 5: APCA^+^ with iron deficiency and borderline vitamin B_12_

#### 5.5.1 ISPAD analysis

In this case, ISPAD generated a very high Pernicious Anemia Probability (PAP = 85.4%). The probability estimate was primarily driven by the combination of corpus-predominant gastric atrophy (OLGA stage II with preserved antral mucosa) and anti-parietal cell antibody (APCA) positivity, despite the absence of anti-intrinsic factor antibodies.

In this case, the borderline serum vitamin B_12_ level increased the probability estimate when combined with concordant histological and immunological parameters. Severe iron deficiency provided additional supportive evidence consistent with autoimmune gastritis and further reinforced the PAP.

Overall, this probabilistic profile illustrates ISPAD’s capacity to identify autoimmune-related pernicious anemia in seronegative AIFA contexts and in the presence of competing deficiencies that may obscure classical hematological patterns.

#### 5.5.2 Clinical correlation

The patient showed clinical and biological improvement following combined parenteral vitamin B_12_ and oral iron supplementation. Neurological complaints, including mild paresthesia and cognitive slowing, resolved despite only borderline baseline vitamin B_12_ concentrations, confirming the diagnosis of pernicious anemia.

This case highlights ISPAD’s ability to support timely diagnosis in complex presentations where borderline biochemical values and dual deficiencies could otherwise delay recognition and treatment of autoimmune-related pernicious anemia.

### 5.6 Case 6: APCA^+^ autoimmune gastritis in type 1 diabetes

#### 5.6.1 ISPAD analysis

ISPAD generated a high Pernicious Anemia Probability (PAP = 77.6%), driven by the combination of corpus-predominant atrophy (OLGA stage II with preserved antral mucosa) and strong anti-parietal cell antibody (APCA) positivity, despite negative anti-intrinsic factor antibodies and normal vitamin B_12_ and ferritin levels.

In this case, histological and immunological concordance constituted the primary diagnostic signal. Normal biochemical parameters exerted only a minimal attenuating effect on the final probability estimate, reflecting ISPAD’s design to detect early autoimmune-related pernicious anemia before overt biochemical deficiency develops.

This behavior illustrates ISPAD’s capacity to model early-stage autoimmune gastritis, particularly in patients with preexisting autoimmune disease, in whom functional impairment may precede measurable vitamin B_12_ deficiency.

#### 5.6.2 Clinical correlation

Given the presence of autoimmune gastritis confirmed by histology, marked hypergastrinemia, APCA positivity, and neurological symptoms, parenteral vitamin B_12_ therapy was initiated despite normal baseline vitamin B_12_ levels. Following treatment, gastrointestinal symptoms and lower-limb paresthesias resolved, supporting the diagnosis.

This case highlights ISPAD’s ability to identify autoimmune-related pernicious anemia in high-risk patients with type 1 diabetes and to support early intervention prior to the development of overt hematological abnormalities or irreversible neurological damage.

### 5.7 Case 7: AIFA^+^ pernicious anemia with gastric cancer comorbidity

#### 5.7.1 ISPAD analysis

In this case, ISPAD generated a very high Pernicious Anemia Probability (PAP = 90.6%). The estimated probability was primarily driven by strong anti-intrinsic factor antibody (AIFA) positivity and severe corpus–fundus–predominant atrophy (OLGA stage III with limited antral involvement), a histopathological pattern highly compatible with autoimmune-related pernicious anemia.

Severe vitamin B_12_ deficiency, together with concomitant iron deficiency, provided additional supportive evidence but did not constitute the primary determinants of the final probability estimate.

In accordance with the overall philosophy of ISPAD, neoplastic disease is not incorporated as a primary diagnostic determinant of pernicious anemia but is interpreted as a potential late complication of the underlying autoimmune gastritis. Accordingly, the system focuses on the diagnostic assessment of pernicious anemia itself, independently of any oncological risk stratification. Within this framework, the presence of gastric cancer neither inflated nor reduced the estimated PAP, thereby preserving diagnostic specificity.

In parallel, the OLGA stage III configuration is associated with an increased risk of gastric neoplasia, as established in the literature, without constituting a marker of established neoplasia. This risk context does not interfere with the probabilistic assessment of pernicious anemia.

#### 5.7.2 Clinical correlation

Parenteral vitamin B_12_ and iron supplementation were initiated, resulting in a rapid hematological response, with hemoglobin levels increasing from 6.8 to 12.9 g/dL within 3 months. This response confirmed the diagnosis of AIFA-positive pernicious anemia despite the coexistence of advanced gastric adenocarcinoma.

This case illustrates ISPAD’s ability to maintain high diagnostic specificity for autoimmune-related pernicious anemia in the presence of major confounding pathology. By prioritizing highly specific immunological markers and compatible histopathological patterns, ISPAD reproduces expert clinical reasoning in complex oncological contexts while remaining strictly focused on the diagnostic assessment of pernicious anemia.

### 5.8 Unified case analysis

**Figure 3:**
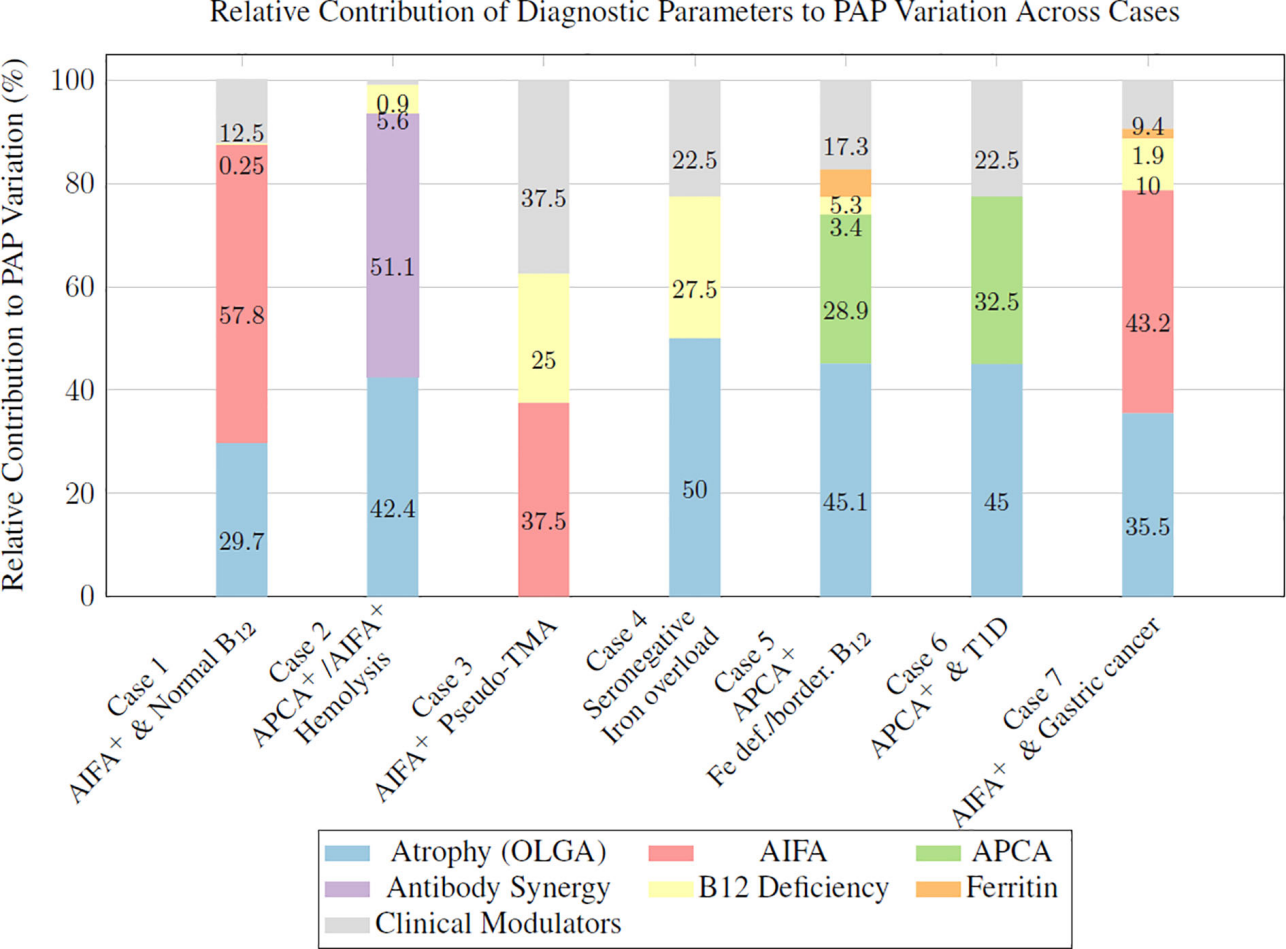
Relative contribution of diagnostic parameters to PAP variation across seven clinically cases. The stacked bar chart illustrates how the ISPAD framework integrates diagnostic parameters according to clinical context, highlighting antibody-dominant profiles (cases 1, 3, and 7), synergistic effects in dual-antibody positivity (case 2), histology-driven patterns in seronegative variants (cases 4–6), and increased influence of functional or biochemical markers in early or masked disease (cases 3 and 5). Values represent normalized estimates of the relative contribution of each parameter to PAP variation, derived from variation-based analysis within the fuzzy inference framework.

To synthesize the diagnostic behavior of ISPAD across heterogeneous clinical contexts, a unified analysis was performed on the seven representative cases (**Figure 3**). The stacked bar representation highlights the relative contribution of histological, immunological, and biochemical parameters to PAP variation across cases, according to the available evidence.

Three main diagnostic patterns emerge. First, antibody-driven profiles (cases 1, 3, and 7) are characterized by dominant AIFA contributions, allowing accurate identification of pernicious anemia despite normal vitamin B_12_ levels, absence of gastric atrophy, or the presence of advanced gastric cancer. These cases illustrate ISPAD’s ability to prioritize highly specific immunological signals over potentially misleading biochemical or structural findings.

Second, histology-driven profiles (cases 4, 5, and 6) demonstrate compensatory reliance on OLGA staging when serological markers are absent or incomplete. In these settings, corpus-predominant atrophy becomes the principal diagnostic anchor, enabling recognition of seronegative or early autoimmune gastritis variants that would escape conventional diagnostic criteria.

Third, synergistic configurations, exemplified by case 2, show how combined antibody positivity and severe fundic atrophy reinforce each other, resulting in maximal diagnostic confidence. Across all cases, ISPAD accommodates complex clinical contexts—including hemolysis, pseudothrombotic presentations, iron overload, autoimmune comorbidity, and malignancy—without excluding diagnoses on the basis of rigid thresholds.

Overall, this unified analysis illustrates the core strength of ISPAD: the formalization of expert clinical reasoning under diagnostic uncertainty. By integrating heterogeneous and sometimes discordant information, the system captures a broad spectrum of pernicious anemia presentations, from classic seropositive forms to atypical, seronegative, and comorbidity-associated variants.

## 6 Limitations, future directions, and regulatory considerations

ISPAD is presented as a methodological proof-of-concept framework designed to formalize diagnostic reasoning in pernicious anemia under conditions of clinical uncertainty. Several limitations should be acknowledged.

First, the present study was not designed as a diagnostic accuracy study. Although ISPAD was explored through systematic parametric analyses and illustrated on seven diagnostically challenging published cases, it has not yet been evaluated on an independent patient cohort. Formal estimates of sensitivity, specificity, predictive values, calibration, or area under the curve are therefore not yet available.

These limitations also imply potential risks in the event of premature clinical deployment. Without external validation, probabilistic outputs could be overinterpreted as definitive diagnostic conclusions or used with unwarranted confidence in heterogeneous real-world settings. In addition, no study has yet assessed the impact of ISPAD on diagnostic confidence, management decisions, clinical workflow, or patient outcomes.

Future work should include retrospective evaluation in larger, well-characterized multicenter cohorts, followed by prospective studies assessing generalizability, comparison with expert judgment and simpler statistical approaches, and potential clinical utility in real-world settings.

Finally, any future translation of ISPAD into a clinical decision-support application would require formal evaluation under applicable regulatory frameworks. In the United States, this would involve assessment under the relevant FDA framework for Clinical Decision Support software; in the European Union, it would require conformity assessment under the Medical Device Regulation (MDR 2017/745). At its current stage, ISPAD is not proposed as a clinically deployable medical device.

## 7 Conclusion

Pernicious anemia represents a late and clinically severe manifestation of autoimmune gastritis. It is characterized by a wide spectrum of non-specific, heterogeneous, and often discordant clinical, histological, immunological, and biochemical findings. This intrinsic complexity frequently delays diagnosis and exposes patients to irreversible neurological or hematological complications.

Addressing this diagnostic uncertainty requires tools capable of formalizing clinical reasoning rather than relying on rigid thresholds or isolated criteria.

In this context, we developed ISPAD, an explainable AI-based probabilistic framework designed to estimate the probability of clinically relevant pernicious anemia in the context of autoimmune gastritis. By integrating a limited but complementary set of routinely available parameters—histological atrophy patterns (OLGA staging), immunological markers (AIFA/APCA), and biochemical indicators of functional deficiency (vitamin B_12_ and ferritin)—while deliberately excluding oncological markers, ISPAD provides a continuous probabilistic output (PAP) intended to reflect structured diagnostic reasoning. The system was co-developed with gastroenterology and digestive oncology specialists to ensure clinical coherence and interpretability.

A key strength of ISPAD lies in its fuzzy inference framework, which allows graded interpretation of partial, conflicting, or incomplete evidence. Rather than categorically excluding pernicious anemia based on a single criterion—such as the absence of atrophy, isolated antibody negativity, or normal vitamin B_12_ levels—the system dynamically adjusts the influence of parameters through its fuzzy inference mechanism. This design enables ISPAD to accommodate early or focal autoimmune gastritis (including OLGA 0 stages), seronegative variants, mixed gastric conditions with limited antral involvement, and situations in which biochemical abnormalities are masked by hemolysis, iron deficiency, or comorbid disease.

System evaluation was conducted in two complementary steps. First, surface plot analyses were performed using simulated but clinically grounded parameter configurations. These simulations were designed to explore how controlled variations in histological severity (OLGA stage and antral involvement), immunological status (AIFA/APCA), and biochemical markers (vitamin B_12_ and ferritin) modulate the estimated probability of pernicious anemia. The resulting surfaces offered an interpretable visualization of the inference mechanism, highlighting the system’s sensitivity to clinically meaningful parameter interactions and its ability to translate pathophysiological patterns into probabilistic diagnostic outputs.

Second, ISPAD was confronted with a curated series of seven real-world clinical cases drawn from the literature, selected to represent a wide spectrum of typical and atypical presentations. This case-based evaluation demonstrates ISPAD’s internal consistency across antibody-dominant forms, histology-driven seronegative cases, dual-deficiency scenarios, early-stage disease without overt atrophy, autoimmune comorbidities, and cancer-associated gastric atrophy. In each context, the system generated coherent probability estimates aligned with reported clinical interpretations and outcomes, including situations in which conventional diagnostic hierarchies would be insufficient or misleading.

Importantly, ISPAD is not intended to replace clinical judgment, histopathological expertise, or established diagnostic workflows. Rather, it provides an interpretable probabilistic framework that formalizes diagnostic reasoning under uncertainty and offers a structured approach to integrating complex diagnostic information. Risk categories generated by ISPAD should therefore be understood as graded estimates that complement, rather than substitute, expert decision-making.

This study has limitations. Validation was performed on retrospective case material, and prospective multicenter studies are needed to confirm generalizability and clinical impact. Additional biomarkers, such as gastrin or pepsinogen levels, may further refine future iterations of the system (ISPAD2), although their exclusion from the current model reflects a deliberate choice to prioritize broadly available and functionally relevant parameters. Future developments may also explore hybrid (44) or type-2 fuzzy logic approaches (73–76) to further enhance uncertainty modeling while preserving interpretability.

Importantly, ISPAD does not redefine diagnostic criteria for pernicious anemia but provides a transparent probabilistic framework that encodes expert clinical reasoning under diagnostic uncertainty and integrates heterogeneous, sometimes discordant diagnostic information.

In conclusion, ISPAD illustrates how an explainable and clinically grounded AI approach contributes to the formalization of the diagnostic reasoning of pernicious anemia. By bridging histology, immunology, and functional biochemistry within an interpretable probabilistic framework, ISPAD offers a structured and interpretable framework for addressing one of the most challenging diagnostic entities in gastroenterology. Further validation on independent cohorts will be required before any clinical application can be considered.

**Table 3:** Summary of ISPAD performance on seven diagnostically challenging real-world cases.

Case	Key diagnostic challenge	Key ISPAD inputs	ISPAD output (PAP)
1	Normal B12 (assay interference)	AIFA^+^, OLGA II, Antrum 0	87.6%
2	Dual seropositivity + hemolysis	AIFA^+^/APCA^+^, OLGA II, Antrum 0	99.1%
3	Pseudothrombotic microangiopathy	AIFA^+^, OLGA 0, Antrum 0	62.5%
4	Seronegative with iron overload	AIFA^−^/APCA^−^, OLGA II, Antrum 0	77.5%
5	Dual deficiency + borderline B12	APCA^+^, OLGA II, Antrum 0	85.4%
6	Autoimmune context (T1D)	APCA^+^, OLGA II, Antrum 0	77.6%
7	Gastric cancer comorbidity	AIFA^+^, OLGA III, Antrum 1	90.6%

## Data Availability

The original contributions presented in the study are included in the article/**Supplementary Material**. Further inquiries can be directed to the corresponding author.
