# Supplementary material for: ISPAD, bridging clinical expertise and AI for autoimmune-related pernicious anemia diagnosis
Source: Front Immunol. 2026 Apr 28;17:1700751. doi: 10.3389/fimmu.2026.1700751 (PMC13162043; doi:10.3389/fimmu.2026.1700751)
Supplement: Supplementary file 1 [file DataSheet1.pdf]

Supplementary Material for ISPAD, Bridging Clinical Expertise and AI for Autoimmune-Related Pernicious Anemia Diagnosis

1 OVERVIEW

This supplementary material contains three main components: (1) detailed membership function parameters for ISPAD input and output variables (Supplementary Tables S1 and S2), (2) the mathematical framework and general inference mechanism underlying the ISPAD fuzzy inference system (Supplementary Text S1), and (3) seven detailed clinical case reports used for system validation (Supplementary Text S2).

2 SUPPLEMENTARY TABLES S1 AND S2: MEMBERSHIP FUNCTION PARAMETERS

2.1 Supplementary Table S1: MF Parameters of Input Variables

Category	N°	Variable	Range	MF	Shape	Parameters
**AG**	1	**OLGA**	Stage 0-4	Stg 0	Triangle-MF	[-0.3, 0, 0.7]
				Stg 1	Triangle-MF	[0.3, 1, 1.7]
				Stg 2	Triangle-MF	[1.3, 2, 2.7]
				Stg 3	Triangle-MF	[2.3, 3, 3.7]
				Stg 4	Triangle-MF	[3.3, 4, 4.7]
	2	**Antrum**	Score 0-3	Scr 0	Triangle-MF	[-0.3, 0, 0.7]
				Scr 1	Triangle-MF	[0.3, 1, 1.7]
				Scr 2	Triangle-MF	[1.3, 2, 2.7]
				Scr 3	Triangle-MF	[2.3, 3, 3.7]
**ABAG**	3	**APCA**	0–500 U/mL	Neg	Trap-MF	[0, 0, 30, 40]
				Pos	Trap-MF	[30, 40, 500, 500]
	4	**AIFA**	0–30 U/mL	Neg	Trap-MF	[0, 0, 7, 10]
				Pos	Trap-MF	[7, 10, 30, 30]
**FIRB**	5	**B12**	0–1,000 pmol/L	Low	Trap-MF	[0, 0, 200, 400]
				High	Trap-MF	[200, 400, 1,000, 1,000]
	6	**Ferritin**	0–300 ng/mL	Low	Trap-MF	[0, 0, 25, 35]
				High	Trap-MF	[25, 35, 300, 300]

Table S1. Detailed membership function parameters for ISPAD input variables. AG = Atrophic Gastritis; ABAG = Autoimmune Biomarkers of Atrophic Gastritis; FIRB = Factors Involved in Red Blood cell formation and iron metabolism. Vector notation: [a, b, c] for triangular MFs; [a, b, c, d] for trapezoidal MFs, where a and d define the outer support, and b and c define the core of the fuzzy set.

2.2 Supplementary Table S2: Gaussian MF Parameters for PAP Output Variable

Variable	Range	MF Type	Class	(μ, σ)
PAP (%)	0–100	Gaussian	EL	(0, 2)
			VVL	(5, 2)
			VL1	(10, 3)
			VL2	(15, 3)
			L1	(25, 4)
			L2	(30, 4)
			LM	(37.5, 5)
			M	(50, 5)
			MH	(62.5, 5)
			H1	(72.5, 5)
			H2	(77.5, 5)
			VH1	(82.5, 4)
			VH2	(87.5, 4)
			VVH	(96, 3)
			EH	(100, 1.5)

Table S2. Gaussian membership function parameters for the PAP output variable. Centers (μ) and standard deviations (σ) are expressed in percentage points. EL = Extremely Low; VVL = Very Very Low; VL1/VL2 = Very Low 1/2; L1/L2 = Low 1/2; LM = Low-Medium transition; M = Medium; MH = Medium-High transition; H1/H2 = High 1/2; VH1/VH2 = Very High 1/2; VVH = Very Very High; EH = Extremely High.

3 SUPPLEMENTARY TEXT S1: FUZZY INFERENCE MECHANISM AND MATHEMATICAL FRAMEWORK

This Supplementary Text details the mathematical framework underlying the ISPAD system. Building upon the parameter definitions and membership functions specified in Supplementary Tables S1 and S2, it outlines the general principles of the fuzzy logic inference engine and the implementation logic used to compute the Pernicious Anemia Probability (PAP). The complete executable implementation of ISPAD, including the full rule base, is provided separately in the native **.fis** file and associated reproducibility package.

3.1 General Fuzzy Inference Framework

To address the complexity and uncertainty inherent in diagnosing pernicious anemia, ISPAD relies on a fuzzy inference system (FIS). This system comprises four interconnected components: the fuzzifier, the rule base, the inference engine, and the defuzzifier [2].

3.1.1 Fuzzifier

The fuzzifier maps crisp scalar inputs (e.g., APCA = 45 U/mL, Ferritin = 57 ng/mL) into degrees of membership in various fuzzy sets. This transformation is achieved through membership functions that assign a degree of belonging between 0 and 1 to each input value. Mathematically, the fuzzification transforms the input vector $\mathbf{x} = [x_1, \ldots, x_p]^T \in X \equiv X_1 \times \cdots \times X_p$ into a fuzzy set F_x in X. This step is essential to activate the inference rules, which are defined in terms of linguistic variables (fuzzy variables) rather than precise numerical values.

3.1.2 Rule Base

The rule base encodes expert knowledge through IF-THEN rules derived from clinical guidelines and domain expertise. Each rule is formulated as a logical implication:

IF $\mathbf{x}$ is A (condition part), **THEN** y is B (action part), where $\mathbf{x} \in X$ and $y \in Y$.

This formulation establishes a relationship between the fuzzy sets A and B, characterized by a membership function $\mu_{A \to B}(\mathbf{x}, y) \in [0, 1]$, which measures the degree of validity of the implication [2].

Consider a system with p inputs $x_1, \ldots, x_p \in X_1 \times \cdots \times X_p$ and one output $y \in Y$, where x_1 is described by Q_1 linguistic terms $\left(T_{x_1} = \{X_{1j}\}_{j=1}^{Q_1}\right)$ and x_p is described by Q_p linguistic terms $\left(T_{x_p} = \{X_{pj}\}_{j=1}^{Q_p}\right)$. The output y is described by Q_y linguistic terms $\left(T_y = \{Y_j\}_{j=1}^{Q_y}\right)$. The lth rule of the base has the following form [2]:

$$R^l : \textbf{IF } x_1 \text{ is } F_1^l \textbf{and } \cdots \textbf{ and } x_p \text{ is } F_p^l \textbf{THEN } y \text{ is } G^l \tag{S1}$$

where M is the total number of rules.

3.1.3 Inference Engine

The inference engine determines how the IF-THEN rules are activated and combined. It matches the fuzzy input sets in X to the fuzzy output set in Y to derive the appropriate output action [2].

The firing strength of the l-th rule, denoted as $\mu_{R^l}(\mathbf{x}, y)$, is calculated as:

$$\mu_{R^l}(\mathbf{x}, y) = \left[\underset{i=1}{\overset{p}{T}} \mu_{F_i^l}(x_i) \right] \star \mu_{G^l}(y) \tag{S2}$$

where T represents the T-norm operator (typically minimum or product) used for conjunction, and $\star$ represents the implication operator [2].

Each rule R^l produces a resultant fuzzy set B^l in the output space Y:

$$\mu_{B^l}(y \mid \mathbf{x}) = \left[\underset{i=1}{\overset{p}{T}} \mu_{F_i^l}(x_i) \right] \star \mu_{G^l}(y), \quad y \in Y \tag{S3}$$

In practical terms, each rule contributes a clipped version of its output membership function, weighted by the minimum activation of its antecedents.

The final aggregated output fuzzy set B is obtained by combining the outputs of all M rules using the S-norm (maximum) operator:

$$\mu_B(y \mid \mathbf{x}) = \max_{l=1}^{M} [\mu_{B^l}(y \mid \mathbf{x})] \tag{S4}$$

In practice, this operation produces the envelope of all rule outputs, preserving the highest activation degree at each output level.

3.1.4 Defuzzifier

The defuzzifier converts the aggregated fuzzy set B into a crisp (precise) scalar value $y(\mathbf{x})$. Among the various defuzzification methods, the Centroid (Center of Gravity) method, which calculates the center of mass of the fuzzy output distribution, is the most used.

$$y(\mathbf{x}) = \frac{\sum_{i=1}^{N} y_i \mu_B(y_i)}{\sum_{i=1}^{N} \mu_B(y_i)} \tag{S5}$$

where N is the number of discretization points y_i in the output domain.

3.2 ISPAD Implementation Specifications

Building on the general framework described above, ISPAD implements a Mamdani-type fuzzy inference system specifically tailored for pernicious anemia diagnosis. In the ISPAD implementation, a *product* T-norm and *Mamdani* implication are used, as detailed below.

The system inputs correspond to the vector $\mathbf{x} = [x_1, \ldots, x_6]^T$ representing the six clinical parameters (OLGA, Antrum, APCA, AIFA, B12, Ferritin). The output y corresponds to the Pernicious Anemia Probability (PAP).

Note : For conceptual clarity, the six ISPAD inputs are presented here by clinical domain (histological, immunological, and biochemical). However, in the executable implementation and reproducibility scripts, the input order is **[OLGA, APCA, AIFA, B12, Ferritin, Antrum]**, and this order must be respected for model evaluation.

The l-th rule ($l = 1, \cdots, M$) follows this structure:

$$\begin{aligned} R^l: \quad & \textbf{IF } OLGA \textbf{ is } F_1^l \textbf{and } Antrum \textbf{ is } F_2^l \quad \textbf{and } APCA \textbf{ is } F_3^l \quad \textbf{and } AIFA \textbf{ is } F_4^l \\ & \textbf{and } B12 \textbf{ is } F_5^l \quad \textbf{and } Ferritin \textbf{ is } F_6^l \quad \textbf{THEN } PAP \textbf{ is } G^l \end{aligned} \tag{S6}$$

Where F_i^l, ($i = 1, \cdots, 6$), the membership functions, are defined as:

- $F_1^l \in \{$Stg 0, ..., Stg IV$\}$ (OLGA stages)
- $F_2^l \in \{$Scr 0, ..., Scr 3$\}$ (Antral atrophy scores)
- $F_3^l, F_4^l \in \{$Neg, Pos$\}$ (Antibody status)
- $F_5^l, F_6^l \in \{$Low, High$\}$ (B12 and Ferritin levels)
- $G^l \in \{$EL, VVL, VL1,VL2, L1, L2, LM, M, MH, H1, H2, VH1, VH2, VVH, EH$\}$ (15 Output PAP classes)

Each rule R^l maps input parameters $\mathbf{x} = [\text{OLGA}, \text{Antrum}, \text{APCA}, \text{AIFA}, \text{B12}, \text{Ferritin}]$ to output risk classes PAP $\in [0, 100]$ via membership functions:

$$\mu_{R^l}(\mathbf{x}, \text{PAP}) = T \begin{bmatrix} \mu_{F_1^l}(\text{OLGA}), \mu_{F_2^l}(\text{Antrum}) \star \\ \mu_{F_3^l}(\text{APCA}), \mu_{F_4^l}(\text{AIFA}), \\ \mu_{F_5^l}(\text{B12}), \mu_{F_6^l}(\text{Ferritin}) \end{bmatrix} \star \mu_{G^l}(\text{PAP}) \tag{S7}$$

The fired rule strength for the lth rule is computed as:

$$\mu_{B^l}(\text{PAP}|\mathbf{x}) = \prod_{i=1}^{6} \mu_{F_i^l}(x_i) \cdot \mu_{G^l}(\text{PAP}) \tag{S8}$$

The fuzzy output set, PAP fuzzy set, is given by combining the rule sets using the *Maximum* operator:

$$\mu_B(\text{PAP} \mid \mathbf{x}) = \max_{l=1}^{M} [\mu_{B^l}(\text{PAP} \mid \mathbf{x})] \tag{S9}$$

The ISPAD system converts the fuzzy output set S9 into a clinically actionable probability score using the centroid method:

$$\text{PAP\%} = \frac{\sum_{j=1}^{100} \text{PAP}_j \cdot \mu_B(\text{PAP}_j)}{\sum_{j=1}^{100} \mu_B(\text{PAP}_j)} \tag{S10}$$

where $\mu_B(\text{PAP}) = \max\limits_{l \in [1,M]} \mu_{B^l}(\text{PAP}|\mathbf{x})$ and $N = 100$ discretization points ensure 1% resolution.

4 SUPPLEMENTARY TEXT S2: DETAILED CLINICAL CASE REPORTS

This supplementary material provides comprehensive clinical narratives and complete diagnostic workups for seven real-world cases used to validate ISPAD. Each case report includes the patient profile, detailed laboratory and diagnostic findings, and the complete ISPAD parameter extraction. For exhaustive clinical context, including treatment outcomes, comorbidities, and extended follow-up, readers are directed to the original published case reports cited in the main text.

Remark 1 : For consistency with the original clinical reports, biochemical values (vitamin B_{12} and ferritin) are presented using the units reported in the source publications. ***Remark 2*** : In ISPAD, ferritin was modeled within the range 0–300 ng/mL. Values above 300 ng/mL were capped at 300 for model evaluation, as hyperferritinemia was outside the scope of the framework. ***Remark 3*** : Regarding OLGA staging, when the exact stage was not explicitly reported in the source publications, the corresponding authors were contacted to obtain the precise classification. In cases where no response was received, the OLGA stage was inferred from the histopathological descriptions provided in the articles, in accordance with standard classification criteria.

4.1 Case 1: AIFA$^+$ with Normal Vitamin B_{12}

4.1.1 Patient Profile

A 49-year-old woman with a medical history of psoriasis vulgaris and chronic depressive disorder was admitted with a 7-day history of asthenia, odynophagia, and low-grade fever. Over the preceding year, she reported progressive depressive symptoms associated with bilateral lower-limb paresthesias. Physical examination revealed pallor and sinus tachycardia (116 bpm), without focal neurological deficits or organomegaly [4].

4.1.2 Laboratory and Diagnostic Findings

Initial laboratory evaluation demonstrated pancytopenia with severe hypoproliferative macrocytic anemia (hemoglobin 7.3 g/dL, mean corpuscular volume 111 fL), leukopenia, thrombocytopenia, and markedly elevated lactate dehydrogenase levels (2,668 IU/L). Peripheral blood smear showed anisocytosis and poikilocytosis, and bone marrow examination revealed moderate trilineage dysplasia.

Serum vitamin B_{12} concentration was within the normal range (387 pmol/L), as were ferritin and folate levels. Anti–intrinsic factor antibodies were strongly positive (92.9 U/mL; approximately 2–3 times the upper limit of normal), whereas anti-parietal cell antibodies were negative. Upper gastrointestinal endoscopy revealed corpus–fundus atrophy with complete antral sparing. Histological examination of gastric biopsies showed chronic non-specific gastritis [4].

4.1.3 ISPAD Input Parameters

- OLGA stage II (moderate corpus–fundus atrophy, inferred from endoscopic and histological findings)
- Antral atrophy score: 0 (preserved antral mucosa)
- AIFA: positive
- APCA: negative
- Vitamin B_{12}: normal (387 pmol/L)
- Ferritin: normal

4.1.4 Clinical Outcome

Following intramuscular cyanocobalamin therapy, the patient exhibited rapid reticulocytosis, normalization of hemoglobin levels, and a marked decrease in lactate dehydrogenase. Neuropsychiatric symptoms improved significantly, and lower-limb paresthesias resolved progressively. The patient remained asymptomatic during long-term follow-up [4].

4.2 Case 2: AIFA$^+$and APCA$^+$ with Autoimmune Hemolytic Anemia

4.2.1 Patient Profile

A 22-year-old African American woman with a medical history of hypertension and iron deficiency anemia presented with a three-week history of progressive fatigue and dyspnea on exertion. She reported heavy menstrual bleeding over the preceding months and had received four units of packed red blood cells one month prior to admission. She had no prior diagnosis of autoimmune disease or neurological symptoms [7].

Physical examination revealed marked pallor, sinus tachycardia (132 beats/min), and icteric sclera. No hepatosplenomegaly or focal neurological deficits were observed.

4.2.2 Laboratory and Diagnostic Findings

Initial laboratory evaluation demonstrated severe normocytic anemia (hemoglobin 4.8 g/dL, mean corpuscular volume 87.7 fL) associated with thrombocytopenia (91,000/μL) and clear biochemical evidence of hemolysis, including markedly elevated lactate dehydrogenase (1,868 IU/L), undetectable haptoglobin (2 mg/dL), and indirect hyperbilirubinemia (1.5 mg/dL). The direct antiglobulin test (IgG) was positive, confirming autoimmune hemolytic anemia.

Vitamin B_{12} concentration was severely reduced (60 pg/mL), with elevated methylmalonic acid and homocysteine levels, confirming functional deficiency. Immunological testing revealed dual positivity for anti–intrinsic factor antibodies (AIFA) and anti-parietal cell antibodies (APCA). Ferritin was elevated (500 ng/mL), consistent with an inflammatory context and recent transfusion history.

Upper gastrointestinal endoscopy demonstrated corpus-predominant gastric atrophy corresponding to OLGA stage II, without antral involvement (antrum score 0). Histological examination confirmed chronic active atrophic gastritis with intestinal metaplasia. Testing for *Helicobacter pylori* was negative.

4.2.3 ISPAD Input Parameters

- OLGA stage II (moderate corpus-predominant atrophy)
- Antral atrophy score: 0 (preserved antral mucosa)
- Anti–intrinsic factor antibodies (AIFA): positive
- Anti-parietal cell antibodies (APCA): positive
- Serum vitamin B_{12}: severely reduced (60 pg/mL)
- Ferritin: elevated (500 ng/mL), in an inflammatory and post-transfusion context

4.2.4 Clinical Outcome

The patient was treated with high-dose corticosteroid therapy for autoimmune hemolytic anemia in combination with parenteral vitamin B_{12} supplementation. This therapeutic approach resulted in rapid hematological improvement, with progressive normalization of hemoglobin levels and resolution of

hemolytic parameters. Vitamin B_{12} replacement was continued on a long-term basis, allowing stabilization of hematological indices and subsequent tapering of corticosteroid therapy.

4.3 Case 3: AIFA^{+} with Pseudothrombotic Microangiopathy

4.3.1 Patient Profile

A 36-year-old black man presented with a two-year history of progressive fatigue and reduced exercise tolerance. He denied gastrointestinal symptoms, weight loss, or neurological complaints. Neurological examination, including cognition, reflexes, vibration, and proprioception, was normal. The absence of gastrointestinal and neurological manifestations contributed to significant diagnostic complexity [6].

4.3.2 Laboratory and Diagnostic Findings

Laboratory evaluation revealed severe anemia (hemoglobin 7.9 g/dL) with associated leukopenia and a platelet count in the low-normal range (157,000/µL). Mean corpuscular volume was elevated (104.4 fL), with an inadequate reticulocyte response (corrected reticulocyte count 1.6%). Markers of hemolysis were prominent, including markedly elevated lactate dehydrogenase (3,988 IU/L) and undetectable haptoglobin (¡30 mg/dL), with schistocytes observed on peripheral blood smear.

Biochemical testing demonstrated severe vitamin B_{12} deficiency (111 pg/mL), with markedly elevated methylmalonic acid (19.69 mM/L) and homocysteine (181.3 µM/L). Iron parameters were preserved (ferritin 380 ng/mL). Serological testing showed positive anti–intrinsic factor antibodies (AIFA), while anti-parietal cell antibodies (APCA) were negative.

Upper gastrointestinal endoscopy revealed no gastric atrophy, corresponding to OLGA stage 0 with preserved antral mucosa. Gastric biopsies showed reactive gastropathy without histological evidence of autoimmune atrophic gastritis.

Based on the combination of severe vitamin B_{12} deficiency, AIFA positivity, and exclusion of alternative causes, a diagnosis of pernicious anemia presenting as pseudothrombotic microangiopathy was established.

4.3.3 ISPAD Input Parameters

- OLGA stage 0 (absence of histologically detectable gastric atrophy)
- Antral atrophy score: 0 (preserved antral mucosa)
- Anti–intrinsic factor antibodies (AIFA): positive
- Anti-parietal cell antibodies (APCA): negative
- Serum vitamin B_{12}: severely reduced (111 pg/mL)
- Ferritin: within normal range (380 ng/mL)

4.3.4 Clinical Outcome

The patient was treated with intramuscular cyanocobalamin therapy. A rapid hematological response was observed, with normalization of hemoglobin levels, resolution of schistocytosis, and recovery of platelet counts within six weeks. Markers of hemolysis progressively normalized. The clinical and biological response confirmed the diagnosis of AIFA-positive pernicious anemia, despite the absence of histologically detectable gastric atrophy at the time of evaluation.

4.4 Case 4: Seronegative (AIFA^{-} APCA^{-}) with Iron Overload

4.4.1 Patient Profile

An 81-year-old Caucasian man was admitted for progressive fatigue associated with severe anemia. His medical history included type 2 diabetes mellitus treated with metformin, ischemic cardiomyopathy, and stage 3 chronic kidney disease. Physical examination revealed vitiligo on both hands, without hepatosplenomegaly or clinical signs of malignancy [5].

4.4.2 Laboratory and Diagnostic Findings

Initial laboratory evaluation showed normocytic, non-regenerative anemia associated with thrombocytopenia and biochemical hemolysis. Vitamin B_{12} levels were undetectable, while serum ferritin and transferrin saturation were markedly elevated. Serological testing for anti-parietal cell antibodies (APCA) and anti–intrinsic factor antibodies (AIFA) was negative.

Bone marrow examination demonstrated megaloblastic changes consistent with vitamin B_{12} deficiency. Upper gastrointestinal endoscopy with systematic biopsies revealed corpus–fundus–predominant atrophic gastritis with preserved antral mucosa, corresponding to OLGA stage II (antrum score 0). *Helicobacter pylori* testing was negative.

Following vitamin B_{12} replacement therapy, hemoglobin and platelet counts normalized within three months, and ferritin levels progressively declined during follow-up.

4.4.3 ISPAD Input Parameters

- OLGA stage II (moderate corpus–fundus atrophy)
- Antral atrophy score: 0 (preserved antral mucosa)
- Anti–intrinsic factor antibodies (AIFA): negative
- Anti-parietal cell antibodies (APCA): negative
- Serum vitamin B_{12}: severely reduced (¡111 pmol/L)
- Ferritin: elevated (537 μg/L), consistent with erythroid-driven iron overload

4.4.4 Clinical Outcome

The patient was treated with parenteral vitamin B_{12} supplementation. Hematological parameters progressively normalized within eight weeks, including recovery of hemoglobin levels and platelet counts. Follow-up demonstrated a gradual decline in ferritin concentrations, reaching 361 μg/L after seven months, consistent with resolution of ineffective erythropoiesis. This clinical and biological response confirmed pernicious anemia in a seronegative context and excluded primary iron overload disorders such as hereditary hemochromatosis.

4.5 Case 5: APCA^{+}with Iron Deficiency and Borderline Vitamin B_{12}

4.5.1 Patient Profile

A 68-year-old Algerian male presented with chronic digestive symptoms and unexplained normocytic anemia (hemoglobin 11.1 g/dL, MCV 84.1 fL). He had no significant comorbidities and was not receiving medications known to interfere with vitamin B_{12} absorption or metabolism. The patient provided explicit consent for the use of his anonymized clinical data.

4.5.2 Laboratory and Diagnostic Findings

Initial laboratory evaluation revealed normocytic anemia associated with severe iron deficiency (ferritin 4 ng/mL) and serum vitamin B_{12} levels (340 pg/mL; reference range 197–771 pg/mL). Despite borderline vitamin B_{12} values, gastric hormonal testing demonstrated marked hypergastrinemia (1031 pg/mL, ¿5× upper limit of normal), consistent with achlorhydria.

Immunological assessment showed positive anti-parietal cell antibodies (APCA 57 U/mL) with negative anti–intrinsic factor antibodies. Upper gastrointestinal endoscopy with systematic biopsies revealed moderate corpus-predominant gastric atrophy (OLGA stage II) with complete antral sparing, consistent with autoimmune gastritis. *Helicobacter pylori* infection was detected 18 months later and was not part of the initial diagnostic evaluation.

4.5.3 ISPAD Input Parameters

- OLGA stage II, antrum score 0
- APCA positive, AIFA negative
- Vitamin B_{12}: 340 pg/mL
- Serum ferritin: 4 ng/mL

4.5.4 Clinical Outcome

The patient was treated with parenteral vitamin B_{12} and oral iron supplementation, resulting in clinical improvement and correction of anemia. Neurological symptoms improved following vitamin B_{12} replacement, supporting the diagnosis of autoimmune-related pernicious anemia despite borderline biochemical findings.

This case illustrates a diagnostically challenging presentation in which competing deficiencies and borderline biomarkers may delay recognition of pernicious anemia, and demonstrates how ISPAD integrates histological, immunological, and biochemical information to generate a clinically coherent diagnostic probability.

4.6 Case 6: APCA$^+$ Autoimmune Gastritis in Type 1 Diabetes

4.6.1 Patient Profile

A 20-year-old African American woman with type 1 diabetes mellitus diagnosed at age 10 (HbA1c 7.8%) was referred for evaluation of a two-month history of nocturnal, non-bloody diarrhea, left lower quadrant abdominal pain, nausea, and lower-extremity paresthesias. Daytime bowel habits were normal. She was followed by neurology for suspected diabetic neuropathy. Physical examination was unremarkable [3].

4.6.2 Laboratory and Diagnostic Findings

Baseline laboratory testing showed normal hematological indices (hemoglobin 12.7 g/dL, MCV 84.1 fL) and normal serum vitamin B_{12} levels (428.9 pmol/L). Iron parameters were within normal ranges (ferritin 46 μg/L, transferrin saturation 16.1%). Immunological testing revealed strong anti-parietal cell antibody positivity (APCA 104.1 AU/mL) with negative anti–intrinsic factor antibodies. Marked hypergastrinemia was present (1,680 pg/mL), consistent with achlorhydria.

Upper gastrointestinal endoscopy demonstrated corpus-predominant atrophy with preserved antral mucosa. Histological examination showed chronic lymphoplasmacytic inflammation with loss of parietal cells, consistent with autoimmune gastritis. *Helicobacter pylori* testing was negative.

4.6.3 ISPAD Input Parameters

- OLGA stage II (moderate corpus–fundus atrophy)
- Antral atrophy score: 0 (preserved antral mucosa)
- Anti-parietal cell antibodies (APCA): positive (104.1 AU/mL)
- Anti–intrinsic factor antibodies (AIFA): negative
- Serum vitamin B_{12}: normal (428.9 pg/mL)
- Ferritin: normal (46 ng/mL)

4.6.4 Clinical Outcome

Given the presence of autoimmune gastritis with corpus-predominant atrophy, marked hypergastrinemia, and neurological symptoms, parenteral vitamin B_{12} supplementation was initiated despite normal baseline serum vitamin B_{12} levels. Gastrointestinal symptoms and lower-limb paresthesias resolved following treatment. The patient remained clinically stable during follow-up, supporting the diagnosis of autoimmune-related pernicious anemia at an early, pre-deficiency stage.

4.7 Case 7: AIFA^{+} Pernicious Anemia with Gastric Cancer Comorbidity

4.7.1 Patient Profile

A 61-year-old Hispanic woman presented with progressive epigastric pain, heartburn, marked asthenia, and unintentional weight loss of approximately 27 kg over six months. She had no known history of autoimmune disease. Physical examination revealed severe pallor without hepatosplenomegaly or lymphadenopathy [1].

4.7.2 Laboratory and Diagnostic Findings

Laboratory evaluation demonstrated severe normocytic anemia (hemoglobin 6.8 g/dL, MCV 81.1 fL), severe vitamin B_{12} deficiency (141 pmol/L), and borderline-low ferritin level (ferritin 11 μg/L; ; local reference range 11–307 ng/mL). Peripheral blood smear showed schistocytes, and lactate dehydrogenase was mildly elevated. Serological testing was positive for anti–intrinsic factor antibodies (AIFA) and negative for anti–parietal cell antibodies.

Upper gastrointestinal endoscopy revealed an extensive ulcerative lesion along the lesser curvature. Histological examination confirmed a poorly differentiated diffuse-type gastric adenocarcinoma associated with moderate-to-severe corpus–fundus atrophy (OLGA stage III) and mild antral involvement. *Helicobacter pylori* testing was negative.

The diagnosis of pernicious anemia was established based on the combination of autoimmune serology, vitamin B_{12} deficiency, and compatible histopathological findings, concurrently with the diagnosis of gastric cancer.

4.7.3 ISPAD Input Parameters

- OLGA stage III (moderate-to-severe corpus–fundus atrophy)
- Antral atrophy score: 1 (mild antral involvement)
- Anti–intrinsic factor antibodies (AIFA): positive
- Anti-parietal cell antibodies (APCA): negative
- Serum vitamin B_{12}: severely reduced (141 pg/mL)

- Ferritin: borderline-low (11 ng/mL)

For Case 7, ferritin was reported as 11 ng/mL, i.e. at the lower limit of the local laboratory reference range (11–307 ng/mL). For model evaluation, this case was encoded using a representative ferritin value of 30 ng/mL, corresponding to the overlap region between the Low and Normal ISPAD ferritin membership functions. The original laboratory value of 11 ng/mL was preserved in the case description. This adjustment is explicitly documented to preserve transparency and reproducibility.

4.7.4 Clinical Outcome

Parenteral vitamin B_{12} and iron supplementation were initiated, resulting in progressive hematological improvement, with hemoglobin increasing from 6.8 g/dL to 12.9 g/dL within three months. The diagnosis of pernicious anemia was confirmed despite the presence of advanced gastric adenocarcinoma. The patient was subsequently referred for oncological management. This case illustrates that autoimmune-related pernicious anemia may coexist with gastric malignancy and that the diagnosis of pernicious anemia remains clinically relevant even in the presence of advanced neoplastic disease.

4.8 Unified Case Analysis

To complement the compositional interpretation provided by the stacked bar representation shown in the main manuscript, the grouped bar chart below facilitates direct comparison of the relative contribution of each diagnostic parameter across cases.

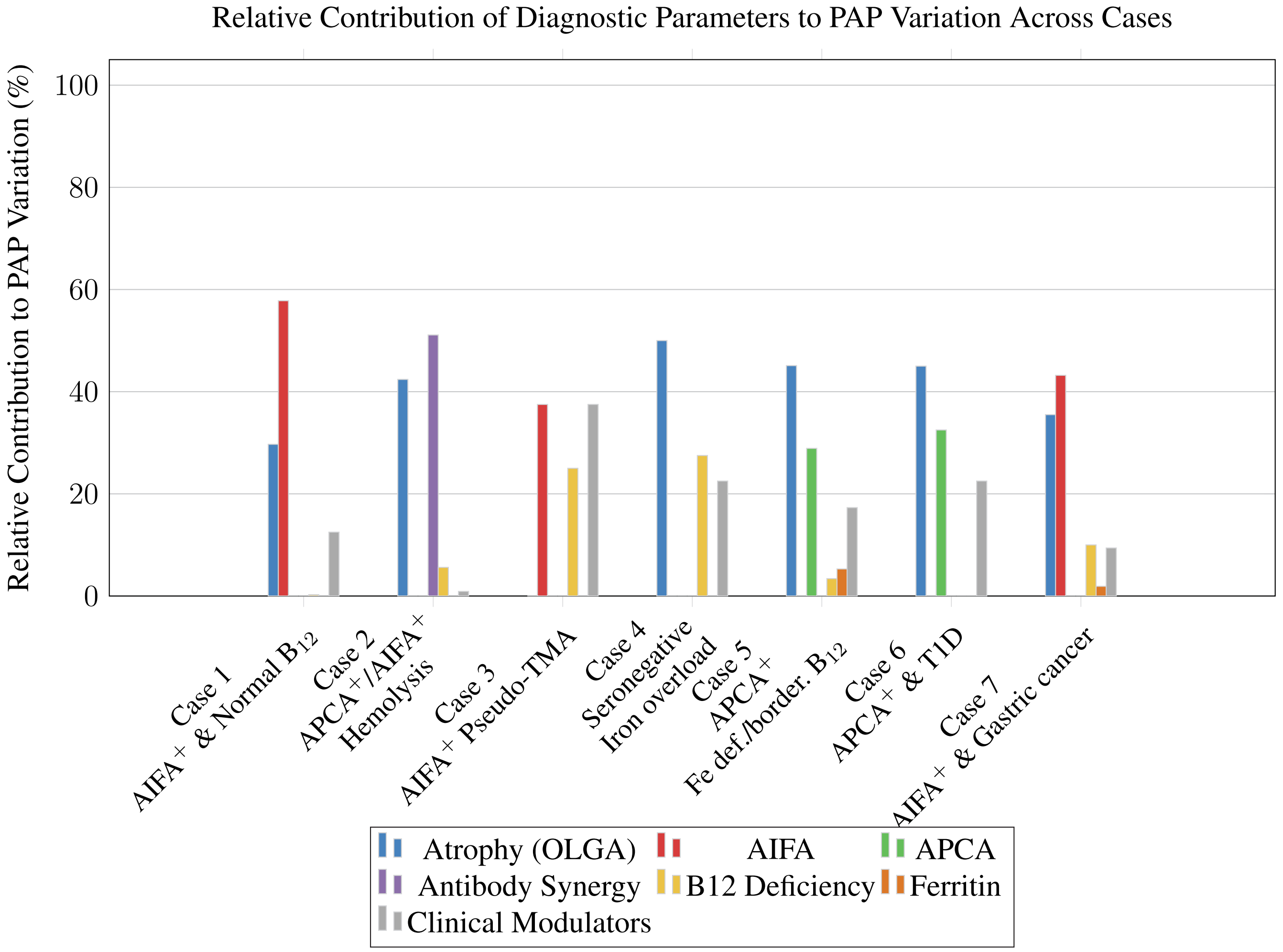

Figure S1. Grouped bar chart showing the relative contribution of each diagnostic parameter to PAP variation across cases. This representation facilitates direct comparison of individual parameter contributions across cases and complements the compositional interpretation provided by the stacked bar chart in the main manuscript.

REFERENCES

[1] S. Kamran, M. K. Dilling, N. A. Parker, J. Alderson, N. D. Tofteland, and Q. V. Truong. Case report: Simultaneously diagnosed gastric adenocarcinoma and pernicious anemia - a classic association. *F1000Research*, 9:604, June 2020.

[2] J. M. Mendel. *Uncertain Rule-Based Fuzzy Logic Systems: Introduction and New Directions*. Prentice-Hall, PTR, Upper Saddle River, NJ, 2001.

[3] T. M. Meyers, P. T. Reeves, J. L. Lombardo, S. K. Anisowicz, N. S. Larson, and P. L. Rogers. Autoimmune gastritis as an unexpected cause of diarrhea in a young adult with type i diabetes: a case report. *Journal of Medical Case Reports*, 17(1):342, July 2023.

[4] J. Tavares, B. Baptista, B. Gonçalves, and A. B. Horta. Pernicious anaemia with normal vitamin b12. *European Journal of Case Reports in Internal Medicine*, 6(2):001045, 2019. Published 2019 Feb 18.

[5] N. Vallet, J. B. Delaye, M. Ropert, A. Foucault, N. Ravalet, S. Deriaz, T. Chalopin, H. Blasco, F. Maillot, O. Hérault, and E. Gyan. Megaloblastic anemia-related iron overload and erythroid regulators: a case report. *Journal of Medical Case Reports*, 15(1):463, Sept. 2021.

[6]K. Veit. Pseudothrombotic microangiopathy and vitamin b12 deficiency in pernicious anemia. *Proceedings (Baylor University Medical Center)*, 30(3):346–347, July 2017.

[7]S. L. Yeruva, R. P. Manchandani, and P. Oneal. Pernicious anemia with autoimmune hemolytic anemia: A case report and literature review. *Case Reports in Hematology*, 2016:7231503, 2016. Epub 2016 Jul 31.
